# The mannose 6-phosphate-binding sites of M6P/IGF2R determine its capacity to suppress matrix invasion by squamous cell carcinoma cells

**DOI:** 10.1042/BJ20121422

**Published:** 2013-03-14

**Authors:** Olivia C. Probst, Evren Karayel, Nicole Schida, Elisabeth Nimmerfall, Elisabeth Hehenberger, Verena Puxbaum, Lukas Mach

**Affiliations:** Department of Applied Genetics and Cell Biology, University of Natural Resources and Life Sciences, Muthgasse 18, A-1190 Vienna, Austria

**Keywords:** cell migration, extracellular matrix, insulin-like growth factor (IGF), lysosome, mannose 6-phosphate (M6P), proteolysis, CD, cathepsin D, CL, cathepsin L, GM130, *cis*-Golgi matrix protein of 130 kDa, HEX, β-*N*-acetylhexosaminidase, IGF, insulin-like growth factor, IGF2R, IGF-II receptor, M6P, mannose 6-phosphate, MPR46, 46 kDa M6P receptor, PDI, protein disulfide isomerase, SCC, squamous cell carcinoma, TGF-β, transforming growth factor β, wt, wild-type

## Abstract

The M6P (mannose 6-phosphate)/IGF2R (insulin-like growth factor II receptor) interacts with a variety of factors that impinge on tumour invasion and metastasis. It has been shown that expression of wild-type M6P/IGF2R reduces the tumorigenic and invasive properties of receptor-deficient SCC-VII squamous cell carcinoma cells. We have now used mutant forms of M6P/IGF2R to assess the relevance of the different ligand-binding sites of the receptor for its biological activities in this cellular system. The results of the present study demonstrate that M6P/IGF2R does not require a functional binding site for insulin-like growth factor II for inhibition of anchorage-independent growth and matrix invasion by SCC-VII cells. In contrast, the simultaneous mutation of both M6P-binding sites is sufficient to impair all cellular functions of the receptor tested. These findings highlight that the interaction between M6P/IGF2R and M6P-modified ligands is not only important for intracellular accumulation of lysosomal enzymes and formation of dense lysosomes, but is also crucial for the ability of the receptor to suppress SCC-VII growth and invasion. The present study also shows that some of the biological activities of M6P/IGF2R in SCC-VII cells strongly depend on a functional M6P-binding site within domain 3, thus providing further evidence for the non-redundant cellular functions of the individual carbohydrate-binding domains of the receptor.

## INTRODUCTION

Intracellular trafficking of most soluble lysosomal proteins depends on their interaction with M6P (mannose 6-phosphate) receptors [1]. Two M6P receptors are known to occur in mammalian cells, the 300 kDa M6P/IGF2R [IGF (insulin-like growth factor) II receptor] and MPR46 (46 kDa M6P receptor). Studies on MPR46- and/or M6P/IGF2R-negative cells have indicated that the simultaneous presence of both receptors is necessary for the optimal delivery of lysosomal enzymes to these compartments [2,3]. However, M6P/IGF2R is generally more efficient than MPR46 in mediating lysosomal enzyme transport, which is at least partly owing to the capacity of M6P/IGF2R to retrieve secreted M6P-modified proteins via the endocytic route [4].

M6P/IGF2R is a multifunctional membrane-associated protein with a repetitive structure consisting of 15 contiguous repeating segments [5]. Its two M6P-binding sites are located in domains 3 and 9. An additional binding site for M6P-*N*-acetylglucosamine residues is located in domain 5 of the protein [6]. Aside of lysosomal enzymes, M6P/IGF2R is supposed to bind also other glycoproteins, such as latent TGF-β (transforming growth factor β) in an M6P-dependent manner. It has been suggested that binding to M6P/IGF2R is required for the pericellular activation of TGF-β [7]. However, the physiological significance of the interaction of M6P/IGF2R with non-lysosomal M6P-modified proteins is still largely unclear.

In addition to interactions mediated by its carbohydrate-binding sites, M6P/IGF2R also has the capacity to bind various proteins by other modes of action, including IGF-II. The principal binding site for IGF-II resides in domain 11 of the receptor [8,9]. It has been shown that mice with a targeted disruption of the M6P/IGF2R locus die perinatally, which can be rescued by simultaneous ablation of the *IGF-II* gene [10]. This indicates that M6P/IGF2R plays a pivotal role in the control of the biological activities of IGF-II. Substantial evidence has been provided that M6P/IGF2R promotes endocytosis and subsequent degradation of IGF-II in lysosomes, thus restricting its bioavailability. Hence M6P/IGF2R counteracts excessive IGF-II signalling through type 1 IGF and insulin receptors rather than directly participating in a signal transduction cascade [11]. It has, however, been proposed that M6P/IGF2R is also capable of acting as a signalling receptor under certain circumstances [12,13].

Given the physiological significance of M6P/IGF2R in the control of important signal transduction events, it is of note that the gene encoding the receptor is frequently mutated in human and animal tumours [14,15]. Evidence has been provided that loss-of-function mutations in M6P/IGF2R contribute to cancer progression, lending support to the notion that this receptor might be a tumour suppressor. Tumour-associated M6P/IGF2R alterations were mainly located in domains 9, 10 and 11 of the receptor [16–19], with this region of the protein hosting one of the two M6P-binding sites and the major site of interaction with IGF-II [5]. The tumour-suppressive potential of M6P/IGF2R is supposed to rely largely on its dampening impact on IGF-II signalling. It has also been suggested that M6P/IGF2R restricts tumour progression by modulation of latent TGF-β activation at the cell surface [20]. However, M6P/IGF2R binds a variety of other factors that could exert an influence on the proliferation, migration and/or invasiveness of tumour cells, including heparanase and cysteine cathepsins [21–23].

Although the growth-suppressive role of M6P/IGF2R is well documented, its impact on tumour invasion and metastasis remains poorly understood. It has been put forward that loss of M6P/IGF2R may promote the invasiveness of malignant tumour cells [24]. Various studies have shown that M6P/IGF2R indeed has the capacity to impede tumour cell migration [25,26]. Interestingly, we have recently found that M6P/IGF2R modulates the invasiveness of liver cells via its capacity to bind M6P-modified proteins [27]. However, the exact mechanisms underlying the anti-invasive properties of M6P/IGF2R in SCC (squamous cell carcinoma) cells remain to be elucidated. Furthermore, it is still unknown whether the individual M6P-binding sites of the receptor serve complementary or redundant functions in the context of anchorage-independent growth and matrix invasion by cancer cells.

We have previously reported that reconstitution of M6P/IGF2R expression in receptor-deficient SCC-VII cells improves the intracellular accumulation of lysosomal enzymes, restores the formation of dense lysosomes and reduces the invasive propensity of the cells [25]. This cellular system was now used to assess the relevance of the different ligand-binding sites of M6P/IGF2R for the biological activities of the receptor by introducing point mutations known to selectively interfere with binding of individual ligands [28,29].

## MATERIALS AND METHODS

### Antibodies

Rabbit antisera raised against bovine M6P/IGF2R, mouse CD (cathepsin D) and mouse proCL (cathepsin L) were kindly provided by Professor Bernard Hoflack (Technical University of Dresden, Dresden, Germany), Professor Regina Pohlmann (University of Münster, Münster, Germany) and Professor Ann H. Erickson (University of North Carolina, Chapel Hill, U.S.A.) respectively. Monoclonal antibodies against rat GM130 (*cis*-Golgi matrix protein of 130 kDa; BD Biosciences) and PDI (protein disulfide isomerase; Stressgen Bioreagents) were obtained from the indicated commercial suppliers.

### Generation of SCC-VII cells expressing human M6P/IGF2R variants

The generation of mutant M6P/IGF2R cDNAs and their insertion into the expression vector pAHygCMV2 has been described previously [27]. The plasmid pAHygCMV2/IGF2R encoding wt (wild-type) human M6P/IGF2R was as described previously [25]. M6P/IGF2R-deficient parental SCC-VII cells [30] were transfected with wt or mutant pAHygCMV2/IGF2R constructs using Lipofectin® (Invitrogen) and then subjected to selection with hygromycin B (Invitrogen). The clones thus obtained were tested for M6P/IGF2R expression as outlined previously for the isolation of SCC-VII/IGF2R wt-1 and wt-2 cells expressing the wt receptor [25,31]. The isolation of mock-transfected SCC-VII cells has been reported previously [25,30].

### Preparation of total cellular membranes

Confluent cell monolayers (1×10^7^ cells) were harvested in 500 μl of PBS containing proteinase inhibitors {1 mM PMSF, 5 μg/ml E-64 [*trans*-epoxysuccinyl-L-leucylamido-(4-guanidino)butane] and 5 μg/ml leupeptin} and disrupted by ultrasonication. Post-nuclear supernatants were obtained by low-speed centrifugation (5 min at 320 ***g***, followed by 5 min at 800 ***g***) and then centrifuged for 60 min at 35000 rev./min (Beckman Coulter 50 Ti rotor) to sediment the membranes.

### Phosphomannan-binding assays

Total cellular membranes were resuspended in 100 μl of binding buffer [0.15 M NaCl, 50 mM imidazole/HCl (pH 7.0) and 0.02% NaN_3_] containing proteinase inhibitors and extracted with 1% (w/v) Triton X-100 for 30 min at 0°C. Aliquots of these membrane extracts corresponding to 150 μg of total protein were diluted 10-fold with binding buffer and incubated with 40 μl of settled phosphomannan–Sepharose beads [25] on an end-over-end mixer for 16 h at 4°C. The beads were then washed five times with 1 ml of binding buffer containing 0.1% Triton X-100. After another wash with 40 μl of 5 mM glucose 6-phosphate (Sigma–Aldrich), the specifically bound material was eluted with 40 μl of 5 mM M6P (Sigma–Aldrich) in the binding buffer.

### IGF-II-binding assays

Total cellular membrane extracts (100 μg of total protein) were diluted 10-fold with binding buffer [0.4 M KCl, 50 mM imidazole/HCl (pH 7.0) and 0.02% NaN_3_ containing 0.1% Triton X-100]. The samples were then incubated either with 1 μg of biotinylated IGF-II (Gropep) or BSA for 16 h at 4°C on an end-over-end mixer, prior to capture of the biotinylated proteins with 40 μl of avidin–Sepharose beads for another 16 h at 4°C [25]. The beads were then washed five times with 1 ml of binding buffer and twice with 1 ml of 10 mM Tris/HCl (pH 6.8). Finally, bound proteins were eluted with SDS/PAGE sample buffer [125 mM Tris/HCl (pH 6.8), 10 mM dithioerythritol, 1% (w/v) SDS, 10% (w/v) glycerol and 0.01% Bromophenol Blue] by incubation for 5 min at 65°C.

### Immunoblotting analysis

Western blotting analysis of M6P/IGF2R was performed after separation by SDS/PAGE (7.5% gel) under reducing conditions. SDS/PAGE (14% gel) was used for the analysis of the other proteins. Samples were denatured for 5 min at 65°C (M6P/IGF2R) or 95°C (other proteins) prior to electrophoresis and blotting on to Hybond-C nitrocellulose membranes (GE Healthcare). Bound primary antibodies were detected with goat anti-rabbit or goat anti-mouse immunoglobulins conjugated to horseradish peroxidase (Jackson ImmunoResearch) and chemiluminescence reagents [30]. Densitometric analysis of immunoblots was done using ImageQuaNT v4.2 software (Molecular Dynamics).

### Subcellular fractionation

Post-nuclear supernatants were obtained and fractionated by density-gradient centrifugation in 18% (v/v) Percoll (GE Healthcare) gradients (initial density of 1.055 g/ml) as described previously [32]. The gradients were divided into ten fractions which were then analysed for their activity of the lysosomal marker enzyme HEX (β-*N-*acetylhexosaminidase). For immunoblot detection of other lysosomal proteins (CD and CL) as well as GM130 (Golgi) and PDI (endoplasmic reticulum), fractions 1–3 (heavy fraction), 4–7 (intermediate fraction) and 8–10 (light fraction) were pooled and extracted as described previously [25,31].

### Proliferation assays

Cells (6×10^5^) were resuspended in 10 ml of culture medium and seeded into 58-cm^2^ dishes. After incubation for 24–72 h, the cultures were harvested by trypsinization and their cell number determined using a Fuchs–Rosenthal chamber.

### Soft agar colony-formation assays

Cells (3×10^3^) were added to 4 ml of medium containing 0.3% agar and seeded into 21-cm^2^ dishes. Colonies obtained after culture for 3 weeks were then counted and their areas determined as described previously [25].

### Matrigel invasion assays

Cells (5×10^4^) were resuspended in 200 μl of serum-free medium containing 0.1% BSA and then seeded on top of Matrigel-coated filters placed in wells filled with 700 μl of conditioned fibroblast medium [25]. After incubation for 24 h at 37°C, the cells were fixed with methanol and then stained with Crystal Violet. After removal of the cells remaining on the upper side of the filter with a cotton swab, the filters were analysed as described previously [25].

### Other methods

Lysosomal enzyme secretion studies and fluorescence microscopy were performed as described previously [25,31,33]. Total protein content was determined either with the Bio-Rad Protein Assay kit (Bio-Rad Laboratories) or the BCA (bicinchoninic acid) Protein Assay Kit (Pierce) as appropriate, using BSA as a standard. Statistical analyses were performed using Student's *t* test, with *P*<0.05 being considered significant.

## RESULTS

### Characterization of mutant M6P/IGF2R variants stably expressed in SCC-VII cells

M6P/IGF2R contains three carbohydrate-binding sites within its extracytoplasmic region, with two of them displaying high affinities for M6P [5]. Studies on bovine M6P/IGF2R have demonstrated an important role for conserved arginine residues in domain 3 (Arg^435^) and domain 9 (Arg^1334^) in M6P binding. Replacement of Arg^435^ and Arg^1334^ (pre-protein numbering) with alanine or charge-conservative lysine residues resulted in complete loss of M6P-binding activity [34]. A structure-based sequence alignment identified Arg^426^ (domain 3) and Arg^1325^ (domain 9) as the corresponding residues in the human receptor [18]. We therefore prepared a Lys^426^/Lys^1325^ double-mutant human M6P/IGF2R construct (M6P/IGF2R Dom3/9^mut^) as well as the respective single-mutant receptor cDNAs, each of the latter still harbouring one functional M6P-binding site. Furthermore, a cDNA variant encoding a point mutation in domain 11 (Ile^1572^→Thr) was generated (M6P/IGF2R Dom11^mut^). This mutation has been previously shown to abrogate binding of IGF-II [8,9].

The mutant M6P/IGF2R cDNAs were then stably expressed in M6P/IGF2R-deficient SCC-VII cells. For each mutant, at least two representative transfectants were chosen for further experiments. The expression levels of the different M6P/IGF2R constructs in the selected cell lines were estimated by immunoblotting (Supplementary Table S1 at http://www.biochemj.org/bj/451/bj4510091add.htm). The receptor content of all selected lines (1.0–2.2 pmol/mg) was well within the physiological range [35] and comparable with that of SCC-VII cells expressing wt M6P/IGF2R (1.0–4.1 pmol/mg) [25].

The subcellular distribution of the mutant M6P/IGF2R variants was investigated by means of fluorescence microscopy. We have previously shown that wt M6P/IGF2R ectopically expressed in SCC-VII cells is present in the perinuclear compartments reminiscent of the Golgi apparatus and Golgi-associated structures such as the *trans*-Golgi network [25]. Immunocytochemical detection of all mutant receptor forms revealed a similar subcellular distribution. This could be verified by complete colocalization with the Golgi marker GM130 (Supplementary Figure S1 at http://www.biochemj.org/bj/451/bj4510091add.htm).

**Figure 1:**
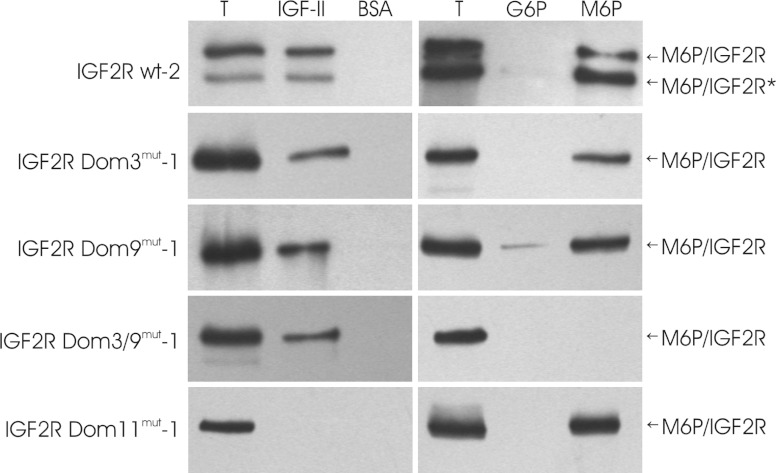
Ligand-binding properties of mutant forms of M6P/IGF2R Left-hand panel, membrane extracts of SCC-VII cells expressing either wt or mutant M6P/IGF2R were incubated with either biotinylated IGF-II or BSA prior to the addition of avidin–Sepharose beads. The bound material was then subjected to immunoblotting analysis with anti-M6P/IGF2R antibodies. Right-hand panel, membrane extracts of SCC-VII cells expressing either wt or mutant receptor variants were incubated with phosphomannan–Sepharose beads. After washing with glucose 6-phosphate (G6P), the specifically bound proteins were eluted with M6P prior to analysis by immunoblotting with anti-M6P/IGF2R antibodies. T, total material applied to the beads. *A C-terminally truncated form of M6P/IGF2R [25].

To investigate whether the mutant M6P/IGF2R forms stably expressed in SCC-VII cells fold and function properly, M6P- and IGF-II-binding studies with membrane extracts of the respective cell lines were performed. The M6P/IGF2R Dom3^mut^ and Dom9^mut^ constructs containing single Arg→Lys point mutations were found to bind IGF-II and phosphomannan, an M6P-containing polysaccharide, like the wt receptor. As anticipated, the double mutant M6P/IGF2R Dom3/9^mut^ did not interact with phosphomannan, but retained its ability to bind IGF-II. Conversely M6P/IGF2R Dom11^mut^ was found to be incapable of interacting with IGF-II, whereas its binding to phosphomannan was not compromized (Figure 1).

### Lysosomal enzyme transport in SCC-VII cells expressing mutant M6P/IGF2R variants

In order to assess whether the M6P/IGF2R mutants are functional in lysosomal enzyme trafficking, the intra- and extra-cellular activities of the lysosomal marker HEX were determined. It should be noted that this assay does not discriminate between direct lysosomal sorting of HEX and reinternalization of secreted enzyme. In our previous study [25], we have already reported that expression of the wt M6P/IGF2R in SCC-VII cells results in a markedly reduced HEX content of the medium (11–24% of the total activity) as compared with parental and mock-transfected cells (59–62%). A similar observation was now made for M6P/IGF2R Dom11^mut^-producing cells (16–21% extracellular HEX), indicating that M6P/IGF2R Dom11^mut^ is fully competent in delivering lysosomal enzymes to their intracellular destination (Table 1).

**Table 1:** Secretion of lysosomal enzymes by parental and transfected SCC-VII cells Cells were cultured for 24 h prior to assaying the cell extracts and conditioned medium for their HEX, CD and CL contents. Results are means±S.E.M. for 3–17 independent experiments. n.a., not analysed.

Cell line	Extracellular activity (% of total)		
	HEX	CD	CL
SCC-VII/IGF2R wt-1†	11±1*	12±4*	73±4
SCC-VII/IGF2R wt-2	18±3*	15±4*	81±2
SCC-VII/IGF2R Dom3^mut^-1	31±3*	16±4*	68±7
SCC-VII/IGF2R Dom3^mut^-2	23±1*	43±1*	74±3
SCC-VII/IGF2R Dom9^mut^-1	20±2*	27±4*	84±7
SCC-VII/IGF2R Dom3/9^mut^-1	38±1*	51±12	87±1
SCC-VII/IGF2R Dom3/9^mut^-2	33±5*	48±2	77±3
SCC-VII/IGF2R Dom3/9^mut^-3	43±3*	52±7	77±6
SCC-VII/IGF2R Dom11^mut^-1	21±3*	n.a.	n.a.
SCC-VII/IGF2R Dom11^mut^-2	16±3*	n.a.	n.a.
SCC-VII mock-transfected†	62±2	67±9	85±3
SCC-VII parental†	59±2	53±5	87±2

*P<0.05, compared with mock-transfected SCC-VII cells.

†HEX data taken from [25].

Interestingly, extracellular accumulation of HEX by SCC-VII cells expressing M6P/IGF2R Dom3^mut^ (23–31%) or Dom9^mut^ (20–24%) is at best moderately increased. Expression of M6P/IGF2R Dom3/9^mut^ led to more HEX being present in the medium (33–43%). However, this value is still significantly lower than that determined for mock-transfected or parental SCC-VII cells (Table 1). To assess whether the improved retention of HEX by SCC-VII cells expressing mutant M6P/IGF2R is indeed related to the ectopic presence of the receptor, the cells were treated with NH_4_Cl, a lysosomotropic amine known to interfere with intracellular sorting of lysosomal enzymes. As previously reported for the wt receptor [25], NH_4_Cl treatment of cells expressing M6P/IGF2R mutants increased the fraction of extracellular HEX to 45–64% (Supplementary Table S2 at http://www.biochemj.org/bj/451/bj4510091add.htm).

We also analysed the intracellular retention of two other lysosomal enzymes, CD and CL, by SCC-VII cells expressing M6P/IGF2R variants with impaired M6P-binding sites. Upon expression of wt M6P/IGF2R, the fraction of CD accumulating in the medium was reduced from 53–67% to 12–15%. The extracellular CD fraction was also lower in SCC-VII cells expressing either M6P/IGF2R mutant with only one functional M6P-binding site (16–43%). In contrast, SCC-VII cells expressing M6P/IGF2R Dom3/9^mut^ accumulated only slightly less CD (48–52%) in the medium than parental and mock-transfected SCC-VII cells (Table 1).

The effect of wt M6P/IGF2R expression on CL retention by SCC-VII cells is much weaker than for HEX and CD [25], which could be related to the existence of an alternative targeting pathway for this enzyme [36]. M6P/IGF2R Dom3^mut^ transfectants were found to deliver even slightly less CL into their medium (68–74%) than cells expressing the wt receptor (73–81%), whereas M6P/IGF2R Dom9^mut^ (84%) and Dom3/9^mut^ cells (77–87%) release almost the same proportion of their CL as their parental and mock-transfected counterparts (85–87%; Table 1).

**Figure 2:**
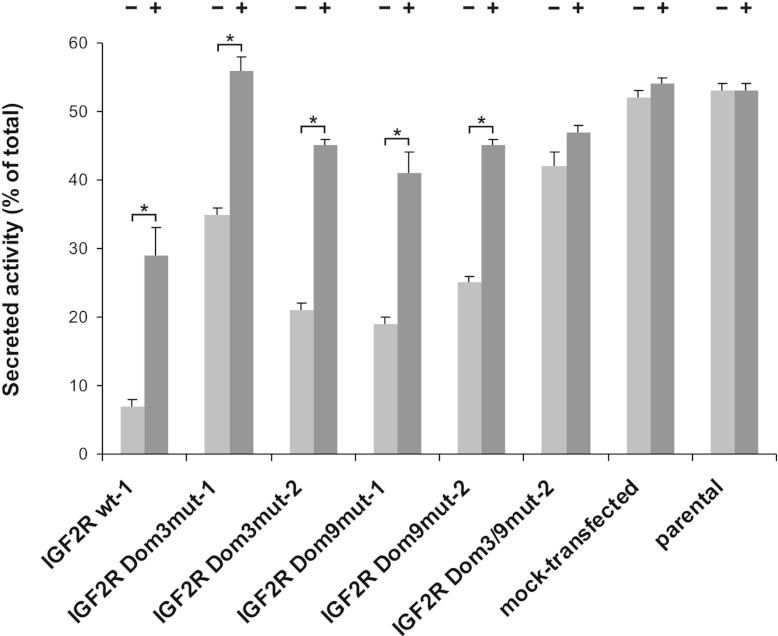
Lysosomal enzyme delivery via the secretion-recapture pathway in SCC-VII cells expressing mutant M6P/IGF2R SCC-VII cells expressing either wt or mutant M6P/IGF2R were cultured for 24 h in the presence or absence of 5 mM M6P. Parental and mock-transfected SCC-VII cells were analysed in parallel. Cell homogenates and conditioned medium were then analysed for their HEX activity. Results are means±S.E.M. for three to four independent experiments. *P<0.05, comparison of treated and untreated cells.

Despite its presence at the cell surface and its recycling between the plasma membrane and intracellular compartments, MPR46 fails to mediate internalization of exogenous ligands. In contrast, M6P/IGF2R is capable of internalizing lysosomal enzymes from secretions [37]. It has been shown that the addition of M6P to the culture medium completely inhibits the reuptake of secreted lysosomal enzymes, without affecting biosynthetic sorting [3]. In the case of SCC-VII cells expressing wt M6P/IGF2R, cultivation in the presence of 5 mM M6P increased the extracellular HEX fraction by 22% of the total enzyme activity. A similar effect was observed for M6P/IGF2R Dom3^mut^ (21–24%) and Dom9^mut^ cells (20–22%). In contrast, the effect of M6P on extracellular HEX accumulation in cultures of M6P/IGF2R Dom3/9^mut^-expressing cells was far less pronounced (5%), as also observed for parental and mock-transfected cells (≤2%). These results demonstrate that the presence of either M6P-binding site is sufficient for normal receptor function in the secretion–recapture pathway (Figure 2).

### M6P/IGF2R-dependent formation of dense lysosomes is impaired by the simultaneous inactivation of its two M6P-binding sites

To investigate the correlation between the expression of mutant M6P/IGF2R and the biogenesis of lysosomes in SCC-VII cells, subcellular fractionation experiments by means of Percoll density-gradient centrifugation were performed. The dense-gradient fractions of mock-transfected SCC-VII cells contain only little HEX activity, as already previously reported for the parental cell line [24,25,31]. In SCC-VII cells expressing wt M6P/IGF2R, a much larger HEX fraction (37–45%) was found to reside in compartments of high buoyant density, mimicking the sedimentation behaviour of the enzyme in normal cells [38]. The same was now observed for M6P/IGF2R Dom11^mut^ cells, with 41% of the total HEX activity detected in the high-density fractions of the gradient. The subcellular distributions of CD and CL were also analysed. In SCC-VII cells expressing the wt receptor, 46–58% of all cellular CD was detected in the high-density region of the gradient, whereas the accumulation of CL in dense compartments was less pronounced (27–34%). Upon expression of M6P/IGF2R Dom11^mut^, only slightly lower amounts of CD (33%) and CL (24%) were found in the high-density gradient fractions. A pronounced accumulation of HEX (36%), CD (58%) and CL (18%) in dense lysosomes was also observed for cells expressing M6P/IGF2R Dom9^mut^. In contrast, M6P/IGF2R Dom3^mut^ cells were found to store far less of their HEX (14–21%), CD (5–12%) and CL (11–14%) in high-density compartments. Even less of the cellular content of HEX (13–15%), CD (4–7%) and CL (8–13%) was present in the high-density fractions of M6P/IGF2R Dom3/9^mut^ cells, which resembles the sedimentation pattern of these enzymes in parental cells (HEX, 16%; CD, 5%; and CL, 8%). These results show that the capacity of M6P/IGF2R to mediate the formation of dense lysosomes in SCC-VII cells strongly depends on the presence of a functional M6P-binding site within domain 3 (Table 2).

**Table 2:** Subcellular fractionation of SCC-VII cells expressing mutant forms of M6P/IGF2R Post-nuclear supernatants were subjected to Percoll density-gradient centrifugation. Fractions 1–3 (dense), 4–7 (intermediate) and 8–10 (light) were then pooled and assayed for their HEX, CD and CL contents. Results are means±S.E.M. for 3–11 independent experiments except for mock-transfected SCC-VII cells (individual results of two fractionations). Markers for the Golgi apparatus (GM130) and the endoplasmic reticulum (PDI) were only detected in the light-gradient fractions.

	Activity in dense fractions (% of total)		
Cell line	HEX	CD	CL
SCC-VII/IGF2R wt-1†	37±1*	46±4*	27±4*
SCC-VII/IGF2R wt-2	45±3*	58±5*	34±3*
SCC-VII/IGF2R Dom3^mut^-1	21±1	5±2	11±2
SCC-VII/IGF2R Dom3^mut^-2	14±3	12±6	14±4
SCC-VII/IGF2R Dom9^mut^-1	36±5*	58±11*	18±2*
SCC-VII/IGF2R Dom3/9^mut^-1	15±2	4±3	10±1
SCC-VII/IGF2R Dom3/9^mut^-2	13±1	5±4	13±1
SCC-VII/IGF2R Dom3/9^mut^-3	13±1	7±3	8±5
SCC-VII/IGF2R Dom11^mut^-1	41±5*	33±7*	24±6*
SCC-VII mock-transfected	17, 17	3	8
SCC-VII parental	16±1	5±4	8±2

*P<0.05, compared with parental SCC-VII cells.

†CD and CL data from [25].

### Simultaneous mutation of both M6P-binding sites abolishes the anti-invasive activity of M6P/IGF2R in SCC-VII cells

**Figure 3:**
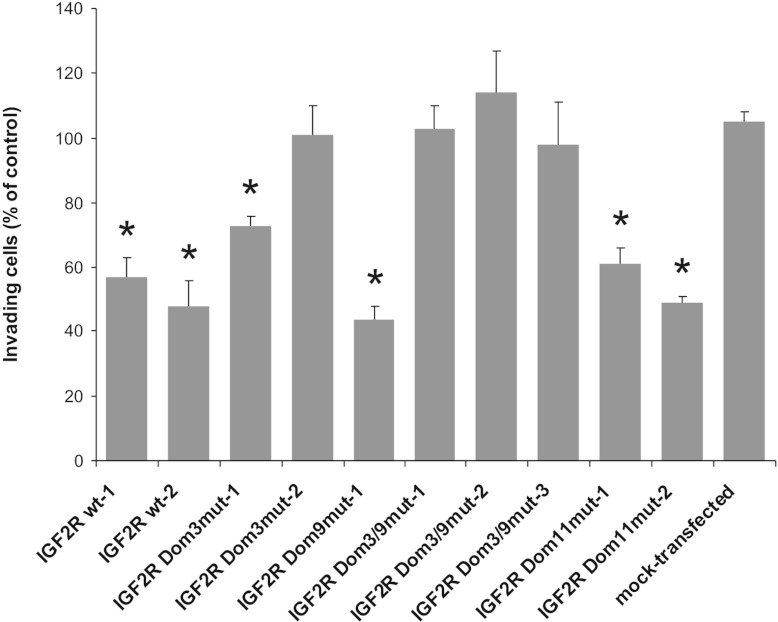
Invasive properties of SCC-VII cells expressing mutant forms of M6P/IGF2R Matrigel invasion assays of SCC-VII cells expressing either wt or mutant M6P/IGF2R were performed using conditioned medium of fibroblasts as a chemoattractant. Mock-transfected cells were analysed in parallel. Parental SCC-VII cells were used as controls (set to 100%). Results are means±S.E.M. for at least three independent experiments. *P<0.05, compared with the mock-transfected cells.

Parental SCC-VII cells display an invasive behaviour when seeded on Matrigel, a reconstituted basement membrane [30]. These invasive properties can be dampened by ectopic expression of wt M6P/IGF2R [25]. The invasiveness of SCC-VII cells expressing M6P/IGF2R Dom11^mut^ was 44–56% lower than that of mock-transfectants, mirroring the effects of wt M6P/IGF2R expression (48–56% reduction). These data provide strong evidence that the presence of a functional binding site for IGF-II is not essential for the anti-invasive capacity of the receptor in SCC-VII cells (Figure 3).

We have also analysed the impact of mutations within the M6P-binding sites on the anti-invasive activity of M6P/IGF2R in SCC-VII cells. As compared with the mock-transfected SCC-VII cells, the invasiveness of M6P/IGF2R Dom9^mut^ cells is similarly reduced (by 61%) as that of SCC-VII cells expressing the wt receptor. M6P/IGF2R Dom3^mut^ expression reduced SCC-VII invasion to a far lesser extent (4–32%), thus providing further evidence that the two M6P-binding sites are not functionally equivalent. Importantly, M6P/IGF2R Dom3/9^mut^ cells exhibited nearly the same invasiveness as mock-transfected SCC-VII cells (≤7% reduction). These data suggest that a functional M6P-binding site within domain 3 is fully sufficient to mediate the anti-invasive properties of the receptor, whereas the M6P-binding site in domain 9 is less effective. Inactivation of both binding sites abolishes the anti-invasive activity of M6P/IGF2R, which suggests that M6P-modified ligands of the receptor play a key role in the migration of SCCs cells across basement membranes (Figure 3).

### The M6P-binding site in domain 3 is of key importance for the growth-suppressive activities of M6P/IGF2R in SCC-VII cells

**Figure 4:**
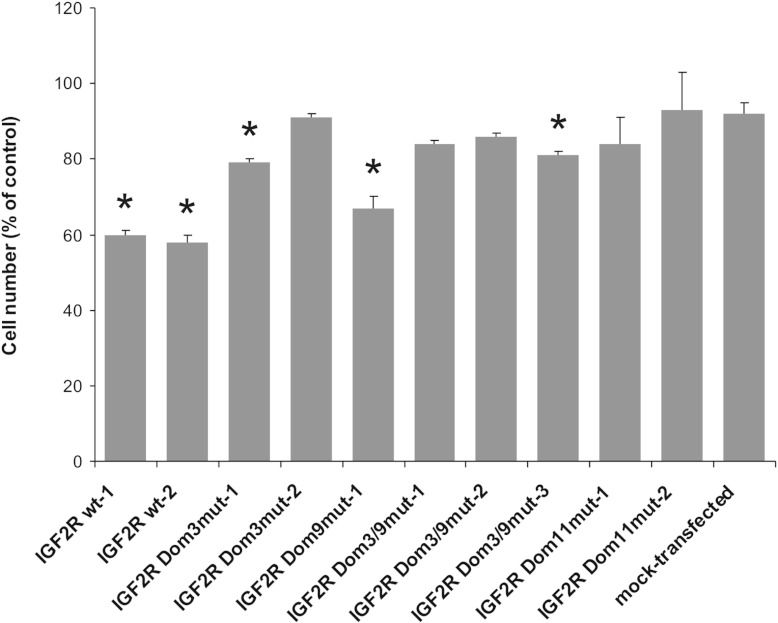
*In vitro* growth of SCC-VII cells transfected with mutant M6P/IGF2R cDNAs SCC-VII cells expressing either wt or mutant M6P/IGF2R were grown for 72 h under standard culture conditions prior to harvesting and cell number determination. Mock-transfected cells were analysed in parallel. Parental SCC-VII cells were used as controls (set to 100%). Results are means±S.E.M. for at least three independent experiments. *P<0.05, compared with the mock-transfected cells.

We have reported previously that expression of wt M6P/IGF2R leads to reduced cell densities in late-stage SCC-VII cultures [25]. To determine the impact of mutant forms of M6P/IGF2R on SCC-VII growth, we followed the cell numbers of the respective cultures over a period of 3 days. During the first 48 h, there were no striking differences between the growth rates of the investigated cell lines. After 72 h of incubation, cultures of SCC-VII cells expressing M6P/IGF2R Dom9^mut^ were found to contain substantially fewer cells than the controls. This phenotype was far less pronounced upon expression of the other M6P-binding site mutants, indicating that M6P/IGF2R-mediated growth arrest depends strongly on the M6P-binding site in domain 3. Interestingly, M6P/IGF2R Dom11^mut^ was similarly ineffective in suppressing the growth of late-stage SCC-VII cultures, suggesting that this activity of the receptor also requires a functional binding site for IGF-II (Figure 4 and Supplementary Table S3 at http://www.biochemj.org/bj/451/bj4510091add.htm).

**Figure 5:**
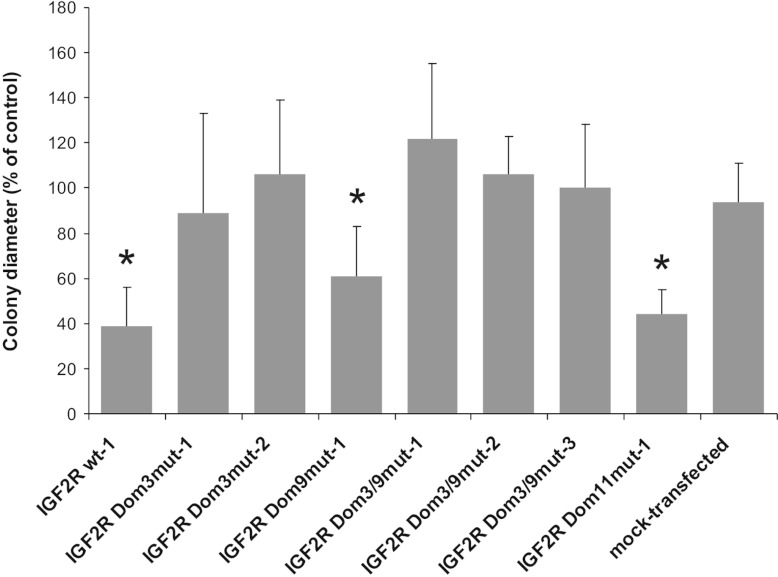
Anchorage-independent growth of SCC-VII cells transfected with mutant M6P/IGF2R cDNAs SCC-VII cells expressing either wt or mutant M6P/IGF2R were cultured for 3 weeks in semi-solid medium before the median diameter of the colonies formed was determined. Mock-transfected cells were analysed in parallel. Parental SCC-VII cells were used as controls (set to 100%). Results are means±S.E.M. for at least three independent experiments. *P<0.05, compared with the mock-transfected cells.

Previous studies have revealed that SCC-VII cells are capable of growing in an anchorage-independent manner when cultured in a semi-solid medium. Although the M6P/IGF2R status did not affect the efficiency of colony formation, colonies formed by SCC-VII cells expressing wt M6P/IGF2R were found to be significantly smaller than those of mock-transfected and parental cells [25]. The same observations were now made for M6P/IGF2R Dom11^mut^-expressing cells, demonstrating that interaction with IGF-II is not required for M6P/IGF2R-mediated restriction of anchorage-independent growth. Interestingly, the median size of colonies formed by M6P/IGF2R Dom3^mut^ and Dom3/9^mut^ transfectants was close to that of parental and mock-transfected cells, whereas colonies of M6P/IGF2R Dom9^mut^-producing cells were only slightly larger than those of SCC-VII cells expressing the wt receptor (Figure 5 and Supplementary Table S4 at http://www.biochemj.org/bj/451/bj4510091add.htm). Taken together, these results indicate that a functional M6P-binding site in domain 3 is important for the capacity of M6P/IGF2R to decrease anchorage-independent growth of SCC-VII cells, thus providing further evidence for the pivotal importance of this binding site for the cellular activities of the receptor.

## DISCUSSION

It is currently believed that the functions of M6P/IGF2R in development and cancer suppression rely mainly on its ability to control the biological activities of IGF-II. Indeed, substantial evidence has been provided that the receptor down-regulates the growth-promoting and anti-apoptotic effects of this potent mitogen, thus preventing developmental abnormalities and tumour formation [10,39]. Recent studies [25–27] have highlighted that M6P/IGF2R also has the capacity to reduce cellular motility and invasiveness. Whereas it has been originally suggested that this is related to altered IGF-II uptake and degradation [40], the anti-invasive activity of the receptor has also been recently linked to its interactions with various proteinases [25,26]. To study the cellular impact of the interactions between M6P/IGF2R and its diverse ligands separately, we have now introduced inactivating mutations into the respective ligand-binding sites prior to stable expression of these mutant receptor cDNAs in M6P/IGF2R-deficient SCC-VII cells, which respond to reconstitution of wt M6P/IGF2R expression with compromized growth and invasiveness [25].

To assess the biological activities of M6P/IGF2R independent of its interaction with IGF-II, we have generated a receptor variant containing a point mutation within the IGF-II-binding site (M6P/IGF2R Dom11^mut^). M6P/IGF2R Dom11^mut^ was found to be less growth-inhibitory than the wt receptor under standard culture conditions, in line with studies performed on LNCaP and PC-3 human prostate cancer cells [41]. However, this M6P/IGF2R mutant is still capable of restricting anchorage-independent growth of SCC-VII cells. Moreover, M6P/IGF2R Dom11^mut^ improves the intracellular accumulation of lysosomal enzymes as efficiently as the wt receptor, thereby quantitatively restoring the formation of dense lysosomes. Matrigel invasion assays revealed that M6P/IGF2R Dom11^mut^ was essentially as anti-invasive as the wt protein. Thus it can be concluded that a functional binding site for IGF-II is not required for the anti-invasive activity of M6P/IGF2R in SCC-VII cells.

The impact of the M6P-binding activity of M6P/IGF2R on cellular growth, motility and invasiveness has not yet been directly studied. However, some cancer-associated M6P/IGF2R mutations have been found to reduce M6P binding [18,19]. To determine whether missense mutations in the two M6P-binding domains affect the growth-inhibitory and anti-invasive activity of M6P/IGF2R, we have produced receptor variants with inactivating point mutations within either of these binding sites. Ligand-binding studies revealed that either of the single-mutant receptors is still able to bind M6P, whereas the double-mutant receptor has lost this capacity. Although it has been reported that the two M6P-binding sites of M6P/IGF2R can differ in their efficiencies of delivering individual acid hydrolases to lysosomes [42], the single mutants proved almost equally effective in mediating the intracellular accumulation of HEX by SCC-VII cells. The same observation was made for CD, but M6P/IGF2R Dom3^mut^ was found to be better than M6P/IGF2R Dom9^mut^ in promoting the retention of CL. Secretion–recapture pathway studies demonstrated that either single mutant matches wt M6P/IGF2R in its capacity to act in the endocytic pathway. However, subcellular fractionation experiments revealed that M6P/IGF2R Dom3^mut^ is far less effective in promoting dense lysosome formation than M6P/IGF2R Dom9^mut^ and the wt receptor. This is possibly explained by the observation that some lysosomal enzymes bind much tighter to domain 3 than to domain 9 [6].

The double mutant M6P/IGF2R Dom3/9^mut^ showed weak residual activity in delivering HEX to lysosomes. This could be owing to the presence of an additional carbohydrate-binding site in domain 5 of the receptor, which exhibits a distinct preference for the biosynthetic intermediate M6P-*N*-acetylglucosamine [6]. It has been reported that domain 5 is capable of mediating the endocytic uptake of a variant of recombinant acid α-glucosidase enriched in such M6P diesters [43]. However, inactivation of both M6P-binding sites almost completely abolished the capacity of the receptor to perform reuptake of HEX secreted by SCC-VII cells. This is probably accounted for by the high efficiency of these cells in uncovering phosphorylated *N*-glycans [31]. Furthermore, M6P/IGF2R Dom3/9^mut^ was found to be unable to support the generation of dense lysosomes in SCC-VII cells, suggesting that the carbohydrate-binding site in domain 5 is also of minor importance for this activity of the receptor.

We have previously reported that expression of wt M6P/IGF2R impedes the growth of SCC-VII cells both *in vitro* and *in vivo* [25]. Analysis of SCC-VII cells expressing mutant receptors now revealed that the M6P-binding site within domain 3 is more important for this activity than the one in domain 9. M6P/IGF2R-mediated growth suppression was almost completely abolished by inactivation of both domains, thus indicating that one or more M6P-modified protein(s) are, when secreted, capable of promoting SCC-VII growth. One candidate for this would be the precursor of the lysosomal aspartic proteinase CD, which has been recently shown to act as a paracrine communicator between cancer and stromal cells in a manner independent of its proteolytic activity [44,45].

The anti-invasive potential of M6P/IGF2R forms lacking individual M6P-binding sites was assessed by Matrigel invasion assays. Although M6P/IGF2R Dom9^mut^ displayed essentially the same anti-invasive behaviour as the wt receptor, M6P/IGF2R Dom3^mut^ was found to be far less effective. Importantly, simultaneous mutation of both M6P-binding sites abrogated the anti-invasive activity of the receptor in SCC-VII cells. These results clearly demonstrate that certain M6P-bearing factors are of substantial relevance for the extent of SCC-VII invasiveness. Thus lack of functional M6P/IGF2R expression possibly favours cancer outgrowth and metastasis largely by enhancing the release of M6P-modified proteins such as lysosomal proteinases and/or other acid hydrolases into the extracellular space. This notion is corroborated by our previous observation that the lysosomal cysteine proteinase cathepsin B contributes to the invasive properties of SCC-VII cells [30]. Previous studies in transgenic mice have provided compelling evidence for multiple functions of cysteine cathepsins such as cathepsin B and CL in tumour invasion and metastasis [22,46,47]. However, the impact of the M6P/IGF2R status on the extracellular accumulation of CL in SCC-VII cultures is only moderate. Hence the pro-invasive M6P-bearing protein(s) secreted by SCC-VII cells still remain to be identified.

## Online data

Supplementary data
