# Supplementary material for: The mannose 6-phosphate-binding sites of M6P/IGF2R determine its capacity to suppress matrix invasion by squamous cell carcinoma cells
Source: Biochem J. 2013 Mar 14;451(Pt 1):91–9. doi: 10.1042/BJ20121422 (PMC3632087; doi:10.1042/BJ20121422)
Supplement: Supplementary data [file bj4510091add.pdf]

BJ Cell

Biochem. J. (2013) **451**, 91–99 (Printed in Great Britain) doi:10.1042/BJ20121422

SUPPLEMENTARY ONLINE DATA

The mannose 6-phosphate-binding sites of M6P/IGF2R determine its capacity to suppress matrix invasion by squamous cell carcinoma cells

Olivia C. PROBST, Evren KARAYEL, Nicole SCHIDA, Elisabeth NIMMERFALL, Elisabeth HEHENBERGER, Verena PUXBAUM and Lukas MACH[1]

Department of Applied Genetics and Cell Biology, University of Natural Resources and Life Sciences, Muthgasse 18, A-1190 Vienna, Austria

Biochemical Journal www.biochemj.org

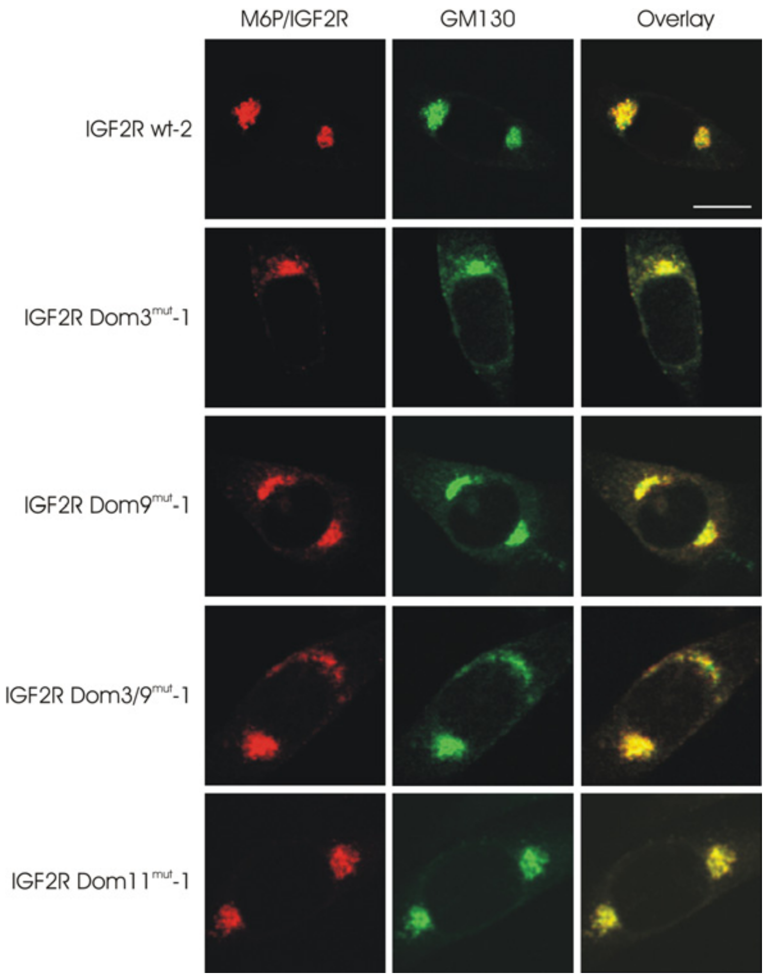

Figure S1 Subcellular localization of mutant forms of M6P/IGF2R

SCC-VII cells stably expressing wt or mutant receptor variants were stained with antibodies against M6P/IGF2R and the Golgi marker GM130. The bound antibodies were then detected with FITC- and Cy3 (indocarbocyanine)-labelled secondary antibodies by confocal laser-scanning microscopy. Colocalization was assessed by merging of the individual images. Scale bar, 10 μm.

[1] To whom correspondence should be addressed (email lukas.mach@boku.ac.at).

© 2013 The Author(s)
The author(s) has paid for this article to be freely available under the terms of the Creative Commons Attribution Licence (CC-BY)(http://creativecommons.org/licenses/by/3.0/) which permits unrestricted use, distribution and reproduction in any medium, provided the original work is properly cited.

Table S1 M6P/IGF2R levels of SCC-VII cells expressing human receptor variants

Results are means ± S.E.M. for at least three determinations. The M6P/IGF2R content of parental and mock-transfected SCC-VII cells is below the detection limit of the assay (0.1 pmol/mg).

Cell line	M6P/IGF2R content (pmol/mg)
SCC-VII/IGF2R wt-1*	2.1 ± 0.1
SCC-VII/IGF2R wt-2*	4.1 ± 0.1
SCC-VII/IGF2R wt-3	1.0 ± 0.1
SCC-VII/IGF2R Dom3^{mut}-1	1.7 ± 0.3
SCC-VII/IGF2R Dom3^{mut}-2	1.4 ± 0.2
SCC-VII/IGF2R Dom9^{mut}-1	1.1 ± 0.1
SCC-VII/IGF2R Dom9^{mut}-2	1.4 ± 0.1
SCC-VII/IGF2R Dom3/9^{mut}-1	1.1 ± 0.1
SCC-VII/IGF2R Dom3/9^{mut}-2	2.2 ± 0.1
SCC-VII/IGF2R Dom3/9^{mut}-3	1.8 ± 0.3
SCC-VII/IGF2R Dom11^{mut}-1	1.4 ± 0.2
SCC-VII/IGF2R Dom11^{mut}-2	1.0 ± 0.1
SCC-VII mock-transfected	<0.1
SCC-VII parental	<0.1

*Taken from [1].

Table S2 Secretion of HEX by parental and transfected SCC-VII cells upon culture for 24 h in the presence or absence of 10 mM NH_4Cl

Results are means ± S.E.M. for three to eight independent experiments.

Cell line	Extracellular activity (% of total)	
	− NH_4Cl	+ NH_4Cl
SCC-VII/IGF2R wt-1†	11 ± 2*	54 ± 4
SCC-VII/IGF2R wt-2†	20 ± 6*	58 ± 8
SCC-VII/IGF2R wt-3	24 ± 2*	54 ± 3
SCC-VII/IGF2R Dom3^{mut}-1	31 ± 3*	46 ± 2
SCC-VII/IGF2R Dom3^{mut}-2	23 ± 1*	45 ± 5
SCC-VII/IGF2R Dom9^{mut}-1	20 ± 2*	58 ± 1
SCC-VII/IGF2R Dom9^{mut}-2	24 ± 1*	50 ± 2
SCC-VII/IGF2R Dom3/9^{mut}-1	38 ± 1*	55 ± 1
SCC-VII/IGF2R Dom3/9^{mut}-2	33 ± 5*	54 ± 3
SCC-VII/IGF2R Dom3/9^{mut}-3	43 ± 3*	52 ± 6
SCC-VII/IGF2R Dom11^{mut}-1	21 ± 3*	52 ± 4
SCC-VII/IGF2R Dom11^{mut}-2	16 ± 3*	64 ± 1
SCC-VII mock-transfected†	62 ± 3	74 ± 1
SCC-VII parental†	60 ± 5	73 ± 2

*$P < 0.05$, compared with mock-transfected SCC-VII cells.
†Taken from [1].

Table S3 *In vitro* growth of SCC-VII cells transfected with mutant M6P/IGF2R cDNAs

Results are means ± S.E.M. for three independent experiments.

Cell line	24 h incubation ($\times 10^5$ cells)	48 h incubation ($\times 10^5$ cells)	72 h incubation ($\times 10^5$ cells)
SCC-VII/IGF2R wt-1	17 ± 1	63 ± 1	95 ± 2*
SCC-VII/IGF2R wt-2	15 ± 1	44 ± 1	92 ± 3*
SCC-VII/IGF2R Dom3^{mut}-1	17 ± 3	62 ± 3	125 ± 1*
SCC-VII/IGF2R Dom3^{mut}-2	13 ± 1	54 ± 1	144 ± 1
SCC-VII/IGF2R Dom9^{mut}-1	19 ± 1	56 ± 1	107 ± 5*
SCC-VII/IGF2R Dom3/9^{mut}-1	19 ± 1	58 ± 1	133 ± 2
SCC-VII/IGF2R Dom3/9^{mut}-2	19 ± 1	62 ± 2	137 ± 1
SCC-VII/IGF2R Dom3/9^{mut}-3	20 ± 1	63 ± 1	129 ± 2*
SCC-VII/IGF2R Dom11^{mut}-1	15 ± 2	57 ± 5	133 ± 11
SCC-VII/IGF2R Dom11^{mut}-2	17 ± 5	58 ± 5	148 ± 16
SCC-VII mock-transfected	19 ± 1	55 ± 1	147 ± 5
SCC-VII parental	18 ± 1	69 ± 1	159 ± 1

*$P < 0.05$, compared with mock-transfected SCC-VII cells.

Table S4 Anchorage-independent growth of SCC-VII cells transfected with mutant M6P/IGF2R cDNAs

Results are means ± S.E.M. for three independent experiments.

Cell line	Colony formation efficiency (%)	Colony diameter	
		Median (mm)	Range (mm)
SCC-VII/IGF2R wt-1†	9 ± 4	0.07 ± 0.03*	0.02–0.25
SCC-VII/IGF2R Dom3^{mut}-1	6 ± 4	0.16 ± 0.08	0.02–0.61
SCC-VII/IGF2R Dom3^{mut}-2	9 ± 4	0.19 ± 0.06	0.02–0.59
SCC-VII/IGF2R Dom9^{mut}-1	8 ± 1	0.11 ± 0.04*	0.02–0.32
SCC-VII/IGF2R Dom3/9^{mut}-1	7 ± 5	0.22 ± 0.06	0.02–0.62
SCC-VII/IGF2R Dom3/9^{mut}-2	14 ± 3	0.19 ± 0.03	0.02–0.64
SCC-VII/IGF2R Dom3/9^{mut}-3	20 ± 4	0.18 ± 0.05	0.02–0.60
SCC-VII/IGF2R Dom11^{mut}-1	15 ± 1	0.08 ± 0.02*	0.02–0.18
SCC-VII mock-transfected†	11 ± 5	0.17 ± 0.03	0.02–0.65
SCC-VII parental†	11 ± 5	0.18 ± 0.04	0.02–0.53

*$P < 0.05$, compared with mock-transfected SCC-VII cells.
†Taken from [1].

REFERENCE

1 Probst, O., Puxbaum, V., Svoboda, B., Leska, V., Stockinger, H., Mikula, M., Mikulits, W. and Mach, L. (2009) The mannose 6-phosphate/insulin-like growth factor II receptor restricts the tumourigenicity and invasiveness of squamous cell carcinoma cells. Int. J. Cancer **124**, 2559–2567

Received 11 September 2012/3 January 2013; accepted 25 January 2013
Published as BJ Immediate Publication 25 January 2013, doi:10.1042/BJ20121422

© 2013 The Author(s)
The author(s) has paid for this article to be freely available under the terms of the Creative Commons Attribution Licence (CC-BY)(http://creativecommons.org/licenses/by/3.0/) which permits unrestricted use, distribution and reproduction in any medium, provided the original work is properly cited.
